# Fecal shedding and tissue infections demonstrate transmission of *Mycobacterium avium* subsp. *paratuberculosis* in group-housed dairy calves

**DOI:** 10.1186/s13567-017-0431-8

**Published:** 2017-04-28

**Authors:** Caroline S. Corbett, Jeroen De Buck, Karin Orsel, Herman W. Barkema

**Affiliations:** Department of Production Animal Health, Faculty of Veterinary Medicine, University of Calgary, Calgary, AB Canada

## Abstract

Current Johne’s disease control programs primarily focus on decreasing transmission of *Mycobacterium avium* subsp. *paratuberculosis* (MAP) from infectious adult cows to susceptible calves. However, potential transmission between calves is largely overlooked. The objective was to determine the extent of MAP infection in calves contact-exposed to infectious penmates. Thirty-two newborn Holstein–Friesian calves were grouped into 7 experimental groups of 4, consisting of 2 inoculated (IN) calves, and 2 contact-exposed (CE) calves, and 1 control pen with 4 non-exposed calves. Calves were group housed for 3 months, with fecal samples were collected 3 times per week, blood and environmental samples weekly, and tissue samples at the end of the trial. The IN calves exited the trial after 3 months of group housing, whereas CE calves were individually housed for an additional 3 months before euthanasia. Control calves were group-housed for the entire trial. All CE and IN calves had MAP-positive fecal samples during the period of group housing; however, fecal shedding had ceased at time of individual housing. All IN calves had MAP-positive tissue samples at necropsy, and 7 (50%) of the CE had positive tissue samples. None of the calves had a humoral immune response, whereas INF-γ responses were detected in all IN calves and 5 (36%) CE calves. In conclusion, new MAP infections occurred due to exposure of infectious penmates to contact calves. Therefore, calf-to-calf transmission is a potential route of uncontrolled transmission on cattle farms.

## Introduction

Johne’s disease (JD) is a chronic, progressive, inflammatory disease in the small intestine of ruminants caused by *Mycobacterium avium* subspecies *paratuberculosis* (MAP). It is well established that MAP infection is widespread in cattle and causes substantial economic losses to dairy producers worldwide [1–3]. Clinical stages of disease cause severe diarrhea and shedding of bacteria into the environment; however, subclinical animals also contribute to the infectious load in the environment and economic losses incurred by the producer, due to reduced milk yield, increased risk of culling and decreased slaughter value [4, 5].

Although vaccines for use in cattle have been developed [6, 7], these vaccines only prevent clinical signs of JD, and there is currently no vaccine available for cattle to prevent infection or shedding of MAP. Therefore, control programs are based on decreasing both the number of new MAP introductions into negative herds and within-herd transmission [2, 3, 8]. The primary route of infection is fecal-oral through ingestion of milk, feed, or water contaminated by infectious animals shedding MAP bacteria in their feces [5, 9, 10]. The assumptions that cows are infectious, calves are susceptible, and calves do not shed until later in life has led to the focus of most control programs interrupting direct and indirect contact of fecal material from infectious adult cows to susceptible young stock [11, 12]. Although the association between JD control programs, management practices, and MAP infections on farms has been well established [2, 12–14], the potential risk of calf–calf transmission is largely overlooked. However, calves can begin shedding as early as 1 month after inoculation [15], calves up to at least 12 months of age are susceptible to MAP infection [16, 17], and a relatively high proportion of young stock on infected farms are shedding MAP in their feces [18, 19]. Although most calves are separated from their dams shortly after birth to prevent transmission, fecal-oral transmission may still be possible during those first hours to days in the calving pen, or even prenatally via intra-uterine transmission [20, 21]. Therefore, group-housing of calves (even though they are isolated from adult cows) may not be an effective practice to eliminate the spread of MAP or prevent new infections.

Recent infection trials have yielded new knowledge that calves inoculated with MAP at an earlier age had more severe tissue lesions [16], and increased fecal shedding was associated with increased numbers of MAP-positive tissue samples [15]. The ability of calves to both infect and become infected has led to several transmission and modelling studies to determine the role of calf-to-calf transmission in causing new infections on farm. However, there have been inconsistent findings regarding the role of calf transmission and its importance for control [17, 22–24]. Furthermore, the extent of infection, subclinical infections, or the ability to suppress an infection, and detecting the signs of infection, all vary depending on several factors, including inoculation dose, immune capabilities, frequency of sampling and individual variability [23, 25, 26]. Therefore, there is a need for an experimental study to examine the extent of infection due to calf-to-calf transmission.

The objective was to determine extent and magnitude of MAP infection in contact-exposed calves resulting from transmission of MAP from inoculated pen-mates, based on fecal shedding and positive tissue samples due to 3 months of group housing.

## Materials and methods

### Calves

Thirty-two newborn Holstein–Friesian bull calves were purchased from 13 Alberta (Canada) dairy farms selected based on annual testing as part of the Alberta Johne’s Disease Initiative [27] and participation in the JD herd health status program in Alberta. All farms had tested negative for at least 4 years using 6 environmental samples and 1 of the following: bacteriological culture of 60 individual fecal samples tested as pooled samples into groups of 5, individual milk ELISA of the whole milking herd, or serum ELISA of the entire herd.

### Nutrition, health and husbandry

All calves were collected immediately after birth (to prevent contamination from fecal material on farm or ingesting colostrum), and transported to the research facility. Nutrition was similar to that described by Mortier et al. [16]. In short, calves were fed 6 L (in 2-L portions) of high-quality colostrum within the first 8 h after birth. Colostrum was collected from 4 of the 13 farms that had tested negative consistently for ≥ 4 years. Starting the 2^nd^ day of their life, calves were fed milk replacer, followed by calf starter (without antimicrobials) and high-quality hay. Calves were gradually weaned by 8 week of age, and had ad libitum access to water and hay (supplemented with concentrates).

Calves were housed in a biosecurity Level 2 facility. The facility included 15 custom-built housing units with waterproof liners to contain all bedding and fecal material. Group-housing pens were 10 × 10 feet and 6 feet tall (3.05 × 3.05 × 1.82 m). Each housing unit consisted of a marked-off area containing the pen, 2 pairs of boots, 2 pairs of coveralls and gloves dedicated specifically for use in the pen within the unit. All personnel were trained to monitor health daily, and to observe strict biosafety and isolation protocols to prevent transmission of MAP between pens by any vectors, e.g. buckets, scoops for feed, personnel, etc. All protocols and the experimental design were approved by the University of Calgary Veterinary Sciences Animal Care Committee (protocol AC14-0168).

### Study design

Calves were assigned to pens based on time of birth and entry into the research facility. The first 14 calves were designated to be inoculated animals (IN), with 2 calves in each of the 7 experimental pens. The next 14 calves to enter the barn were assigned as contact-exposed (CE) and individually housed temporarily in separate pens from the IN calves. The last 4 calves to enter the barn were designated as the control group, and placed together in the control pen. At 2 weeks of age, the IN calves in each pen were inoculated over 2 consecutive days. After 2 weeks (to allow the inoculum to pass through the calves), pens were relined with new liners and bedded with fresh shavings and straw. Calves designated as CE had to reach a minimum of 1 week of age with no health complications to ensure that they could drink from a bucket without assistance, and that only healthy calves were added to the study. When both CE calves entering the same pen reached a minimum 1 week of age, they were placed into the clean, re-lined experimental pen with the IN calves. Four calves (2 IN and 2 CE) were then group-housed for 3 months following the first day of group housing. The IN calves were euthanized and necropsied after 3 months of group housing. The CE calves were then individually housed in relined and clean pens for an additional 3 months. All 4 control calves were group housed (1 pen) for the entirety of the study.

### Inoculum

The inoculum was a virulent MAP cattle type strain from a clinical JD case in Alberta (Cow 69) [16]. In short, a culture was prepared in 7H9/mycobactin/OADC liquid broth, from a first passage frozen stock and quantified using a combination of optical density (OD) at 600 nm, the wet weight method, and qPCR, as described [28]. Once culture grew to a concentration of 5 × 10^8^ CFU/mL, 1 mL aliquots were frozen at −80 °C until 1 week prior to inoculation. Before each inoculation, 1 tube was thawed and suspended in 50 mL 7H9 broth for 1 week, during which time inoculum was tested for contamination. 2.5 × 10^8^ CFU’s was quantified using the wet weight method, diluted in 20 mL of broth, placed in a 20-mL syringe and transported to the research facility. Calves were allowed to suckle the syringe containing the inoculum and it was expelled at the root of the tongue (on 2 consecutive days).

### Fecal sampling and culture

Fecal samples were collected daily for 14 days following inoculation of IN calves to ensure viability of the inoculum, and monitor passive shedding. As of 14 days after inoculation, shedding was attributed to active MAP infection. For the remainder of the trial, fecal samples from each calf were collected three times/week during group housing for all calves. Following group housing, when calves were housed individually, fecal samples were collected weekly from CE calves for the remainder of the trial. Samples were stored at 4 °C until processing, which occurred within 7 days after collection.

All samples were processed using a modified TREK ESP II culture media (TREK para-JEM^®^; TREK Diagnostic Systems, Cleveland, OH, USA) with subsequent F57-specific qPCR, as described [15]. Briefly, 2 g of fecal sample was thoroughly mixed with 30 mL of distilled water and left to settle for 30 min. Then, 5 mL of supernatant was transferred to 25 mL of a 0.9% hexadecylpyridinium chloride (HPC) half-strength brain heart infusion (BHI) solution for decontamination. Samples were then incubated for 24 h at 37 °C, followed by centrifugation at 3000 × *g* for 20 min, and the pellet re-suspended in a mixture of antibiotic solution (AS; para-Jem^®^), water, and full strength BHI. Tubes were incubated again for 24 h at 37 °C and then 1 mL was added to liquid culture medium in TREK para-JEM^®^ culture bottles (TREK Diagnostic Systems, Cleveland, OH, USA) and incubated at 37 °C for 49 days.

### Environmental sampling and culture

Environmental samples were collected once per week from each pen for the duration of the trial. Samples were collected from 5 locations within the pen, and mixed together, resulting in 1 composite sample from each pen. Samples were collected from the surface of the bed pack (individual piles of feces were avoided). Samples were stored at 4 °C until processing, and were subjected to the same protocol (described above) as fecal samples.

### Necropsies and tissue cultures

The IN calves were euthanized after 3 months of group housing at 4 months of age by intravenous injection of barbiturate (Euthanyl Forte^®^, DIN 00241326, Bimeda-MTC Animal Health Inc., Cambridge, ON, Canada), whereas CE were euthanized at 6 months of age, after an additional 3 months of individual housing. Control calves were euthanized last, after all other animals had exited the trial. Necropsies were performed immediately after euthanasia. No other ruminants were examined in the pathology room during necropsies, and the pathology room and tables were thoroughly cleaned and disinfected before and after each necropsy. Thirteen tissue samples were collected from each calf, including two sections of the duodenum, the ileum (including ileal-cecal valve), three sections of jejunum, and spleen. All associated lymph nodes with each gastrointestinal tract section were also collected, as well as the inguinal lymph nodes. Sample locations were marked and isolated with zip ties prior to collection (to prevent movement of intestinal contents). A new set of disinfected instruments and a new pair of gloves was used for collection of each new sample to prevent cross contamination, and PBS was used to rinse fecal content from intestinal tissues.

Samples were transported to the laboratory, and processed immediately on the same day using a modified version of a previous protocol [16]. Briefly, 2.5 g of tissue was dissociated using gentleMACS M tubes (Miltenyi Biotech Inc, Auburn, CA, USA) in 10 mL 0.5% triton x-100 PBS solution. Samples were then transferred to a falcon tube and centrifuged at 4700 × *g* for 15 min and the pellet re-suspended in 25 mL of 0.75% HPC, ½ strength BHI, 4-mm sterile glass beads and vortexed vigorously for 1–2 min. Samples were then incubated at 37 °C for 3 h, before centrifugation at 4700 × *g* for 15 min. The pellet was then re-suspended in 3 mL of antibiotic brew (paraJEM^®^) and incubated overnight, and 1 mL added to paraJEM^®^ culture bottles and incubated at 37 °C for 49 days.

### qPCR procedure

Following liquid culture of fecal and tissue samples, DNA was extracted as described [29]. A duplex qPCR targeting the MAP-specific F57 region and an internal amplification control (IAC) was performed, with primers, probes, and IAC sequences identical to those described [30]. Amplification conditions for qPCR were as follows: 50 °C for 2 min, 95 °C for 20 s to allow for initial denaturation, then 42 cycles of 95 °C for 30 s and 59 °C for 30 s. Samples were considered positive when the cycle threshold (CT) value was < 40.

### Blood sampling, IFN-γ release assay and ELISA

Blood samples were collected weekly from the jugular vein of all calves, alternating between sides. Whole blood was transported to the lab in heated coolers with hot water bottles (25–35 °C), and processed within 2 h for detection of IFN-γ release, as described [31]. Briefly, each sample of whole blood was treated with 100 μL avium Purified Protein Derivative (aPPD; 0.3 mg/mL; Canadian Food Inspection Agency, Ottawa, ON, Canada), 100 μL of pokeweed mitogen (positive stimulation control; 0.3 mg/mL; Sigma-Aldrich Canada Co., Oakville, ON, Canada), and 100 μL sterile PBS (negative stimulation control). Following overnight incubation at 37 °C, serum was collected after centrifugation and stored at −20 °C until all samples were collected and assayed using the sandwich ELISA BOVIGAM^®^ (Prionics, La Vista, NE, USA). Inclusion criteria and interpretation of the IFN-γ release assay were as described [15, 32]. Consequently, observations were excluded from analysis if negative assay controls were < 0.25, the difference between the positive and negative assay controls was < 0.45 or there was a difference of < 0.20 between the negative stimulation and negative assay control. These criteria resulted in only 12 samples being excluded from the study. The % IFN-γ was calculated as follows [31, 32]:

$$\left[ {\left( {{\text{PPD Johnin}} - {\text{negative assay control}}} \right)/\left( {{\text{positive}} - {\text{ negative assay control}}} \right)} \right] \times 100.$$

Serum was collected for antibody testing following centrifugation and stored at −20 °C until antibody ELISA testing was performed, based on manufacturer’s directions (IDEXX Laboratories Inc.), with analysis as described [33]. Briefly, sample results were expressed as a proportion of the positive control corrected for the negative control (S/P ratio), and a ratio ≥ 60 was considered positive.

### Data and statistical analyses

All statistical analyses were performed using STATA 11.2 (StataCorp LP, College Station, TX, USA). For all analyses, *P* < 0.05 was considered significant.

To define shedding events, isolated fecal culture-positive samples (sample collected week prior and subsequent week were negative), and groups of positive samples in which a positive sample was immediately followed by a subsequent positive fecal sample(s), were categorized as a single shedding event. Difference in mean number of fecal positive samples and shedding events, and length of shedding period between IN and CE calves was evaluated using a Student’s *t* test. The average length of events for IN and CE calves was calculated separately. Calves were also separated into fecal shedding categories based on the number of positive samples during group housing, where: 1 = calves with 0–4 positive fecal samples; 2 = calves with 5–9 positive fecal samples; 3 = calves 10–14 positive fecal samples; and 4 = calves with ≥ 15 positive fecal samples of all 38 samples collected during group housing.

The INF-γ results were dichotomized using a cutoff of 100% IFN-γ by calculating the average of presumed negative calves (control calves) + 1.96 the standard deviation [34]. All samples with a value of  % IFN-γ exceeding 100, immediately followed by a sample below 100% IFN-γ, were considered false-positive spikes and removed from analysis (28 samples were excluded).

Differences in fecal shedding category, tissue culture and IFN-γ results between IN and CE calves, as well as the association between having at least 1 positive IFN-γ sample and having at least 1 tissue-positive sample, were evaluated using a Fisher’s Exact test.

## Results

### Tissue culture

**Figure 1:**
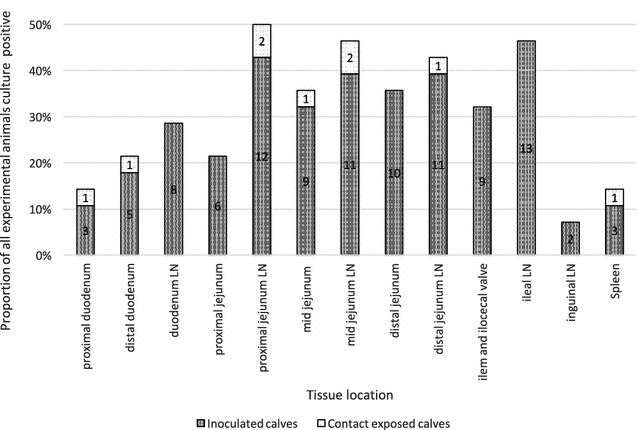
**Proportion of calves with** ***Mycobacterium avium*** **subspecies** ***paratuberculosis*** **culture-positive tissue samples per location.** The y-axis displays the proportion that 1 particular tissue was positive over all calves exposed to MAP, whereas the x-axis indicates tissue location. Numbers indicate number of calves with a culture-positive tissue in the particular location. LN, lymph node.

All IN calves had at least 3 MAP-positive tissue cultures (range 3–11), whereas 7 (50%) of the CE calves had at least 1 MAP-positive tissue culture, but no more than 2 positive tissue samples (Table 1; Figure 1; *p* < 0.001). None of the control calves had positive tissue cultures.

**Table 1:** **Number of** ***Mycobacterium avium*** **subspecies** ***paratuberculosis*** **fecal culture and tissue culture-positive calves in the 3 experimental groups**

Calf status	Fecal culture^a^				Tissue culture^b^			
	1	2	3	4	0	1	2	3
Inoculated (*n* = 14)	0	1	3	10	0	1	4	9
Contact-exposed (*n* = 14)	3	10	1	0	7	7	0	0
Control (*n* = 4)	4	0	0	0	4	0	0	0

^a^1, calves with 0–4 fecal culture positive samples; 2, calves with 5–9 fecal culture-positive samples; 3, calves with 10–14 culture-positive samples; and 4, calves with ≥ 15 fecal culture-positive samples.

^b^0, calves with 0 tissue culture-positive samples; 1, calves with 1–3 tissue culture-positive samples; 2, calves with 4–6 tissue culture-positive samples; and 3, calves with > 6 tissue culture-positive samples.

All tissue locations were positive in at least 2 IN calves. No location was MAP culture-positive in all calves; however, lymph nodes associated with the jejunum were most frequently MAP-positive, especially among tissue-positive CE calves (Figure 1).

### Immune responses

For all calves, all samples were antibody ELISA-negative, except for 2 pre-infection samples of Calves 15 and 16 that tested positive on 1 occasion before testing negative for the remainder of the study, and this may be due to the transfer of maternal antibodies absorbed from colostrum intake.

**Figure 2:**
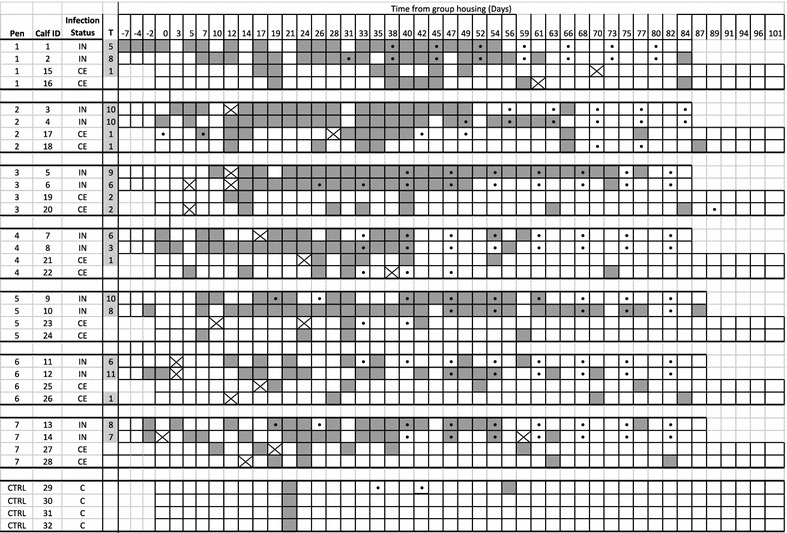
***Mycobacterium avium*** **subspecies** ***paratuberculosis*** **fecal culture, tissue culture and INF-γ results for individual calves per pen.** A solid dark grey box indicates a positive fecal culture by F57-specific qPCR, a white box indicates a negative culture sample and box with a cross indicates a missing sample. “T” indicates the culture results for tissue samples, boxes shaded light grey indicate positive samples, and number of samples out of 13 that tested positive. Dots indicate blood samples that tested positive for IFN-γ (based on 100% INF-γ cut-off).

All IN calves had at least 5 positive IFN-γ responses, whereas 5 (36%) CE calves had at least 1 positive IFN-γ response (Figure 2). Additionally, 1 control calf (C29) had 2 positive INF-γ samples at 2 consecutive time points (35 and 42 days after beginning of group housing) during the experimental trial.

The IN calves started to have positive INF-γ samples on average 55 days after inoculation (41 days after the start of group housing), with the earliest and latest being 33 and 73 days after inoculation, respectively. Eight (64%) of the IN calves had their first INF-γ response on or after 56 days following inoculation and the average interval after the start of group housing for the CE calves to have an INF-γ response was 45 days, with the earliest being the day of exposure, and the latest day 89 of group housing (*p* = 0.32); however, 3 (60%) of these CE calves had positive INF-γ response on or before 33 days after the start of group housing.

Among CE calves, the tissue sample outcome was not associated with the INF-γ result (*p* = 0.58).

### Fecal shedding of MAP

No calves fecally shed MAP prior to exposure (inoculation for IN, and group housing for CE). Positive fecal samples were detected consistently in all IN animals for at least 7 days after inoculation (14 days before group housing), and as many as 10 days. The first positive fecal sample collected from a CE calf occurred 5 days after the start of group housing, whereas the latest first shedding event was detected 31 days after the start of group housing (Figure 2). All IN and CE calves had at least 2 positive fecal samples during the 3 months of group housing (Figure 2); however, all fecal samples from CE calves were negative after group housing ended and they were housed individually (Figure 2). Fecal samples from control calves were culture-negative for all time points, except for day 21 after the start of group housing at which time all calves had a positive sample (Figure 2), whereas Calf 29 also had 1 additional positive sample on day 56.

Mean number of shedding events was 5.6 (95% CI 4.6–6.7) and 4.1 (95% CI 3.4–4.9) in IN and CE calves, respectively (*p* = 0.02). Mean number of positive samples was 17.6 (95% CI 14.4–20.9) and 5.1 (95% CI 3.9–6.2) in IN and CE calves, respectively (*p* < 0.001). Thirteen (93%) of the IN calves were categorized into Groups 3 and 4, whereas 13 (93%) CE calves were categorized into Groups 1 and 2 (Table 1). Average length of a shedding event among the IN calves was 7.5 days, ranging from 2 to 54 days, whereas an average shedding event of CE calves lasted 2.9 days, ranging from 2 to 9 days (*p* = 0.001; Figure 2).

### Environmental culture

All experimental pens had at least 2 MAP culture-positive environmental samples with a maximum of 7 of the weekly 12 samples collected during group housing (Table 2). The earliest environmental samples were culture-positive was 3 days after the start of group housing, and the latest first positive sample was collected 28 days after the start of group housing. The control pen had no environmental culture-positive samples. There were no significant correlations between positive environmental samples and shedding in all calves (*p* = 0.47), among CE shedding (*p* = 0.30), IN shedding (*p* = 0.41), or positive tissue samples (*p* = 0.49).

**Table 2:** ***Mycobacterium avium*** **subspecies** ***paratuberculosis*** **environmental sampling results for all pens during group housing**

Group housing (week)	Pen ID								Total
	1	2	3	4	5	6	7	Controls	
1	−	+	−	+	+	+	−	−	4
2	−	−	+	−	−	−	–	−	1
3	−	−	−	+	+	−	+	−	3
4	+	+	−	−	−	−	+	−	3
5	+	+	+	+	−	+	+	−	6
6	+	+	+	−	−	−	+	−	4
7	+	+	+	−	−	+	+	−	5
8	+	−	+	−	−	−	+	−	3
9	−	−	+	−	−	+	+	−	3
10	−	−	−	−	−	−	−	−	0
11	−	−	−	−	−	−	−	−	0
12	−	−	−	−	−	−	−	−	0
Total	5	5	6	3	2	4	7	0	32

## Discussion

In 5 of the 7 experimental pens, at least 1 CE calf had MAP-positive tissue samples, indicating infection caused by exposure to IN animals in the group pen. In total, 50% of CE calves had MAP-positive tissue samples, 5 (36%) had a positive INF-γ response, and all CE calves shed MAP during group housing. However, there was no association between INF-γ, or MAP-positive tissue results among CE calves. The majority of MAP-positive tissue samples from all calves were isolated from the ileum, jejunum, and adjacent lymph nodes, consistent with other studies [16, 35–37].

A low to moderate inoculation dose was chosen to be representative of natural exposure [35, 38, 39]; however, the inoculation protocol will likely have led to a difference in MAP dose between IN and CE calves, as IN calves were artificially infected. Because CE calves were infected by exposure to a contaminated environment and infectious animals, the dose and number of exposure events among CE calves cannot be directly determined. A higher inoculation dose results in a higher number of MAP-positive tissues, and was likely the origin of differences between IN and CE calves in number of positive tissue samples detected [15, 16, 35, 39]. Although there is little known regarding mechanisms of MAP shedding, it is generally agreed that shedding occurs as a result of MAP excretion towards the intestinal lumen [40]. As the majority of positive tissue samples in CE calves were located in the LN associated with the jejunum, jejunal and ileal tissue samples were mostly negative in CE calves, this may explain cessation of shedding following individual housing, as MAP was not detected where shedding is hypothesized to occur [40]. Additionally, CE calves had considerably fewer positive tissue samples than IN calves, and an increased number of culture-positive tissue samples has been associated with an increased frequency of MAP shedding [15]; therefore, the extent of infection among the CE calves may have been less than the IN calves, leading to less frequent fecal shedding.

All calves had MAP-positive fecal samples during group housing, indicating exposure and risk for infection to all CE calves. Although all CE and IN calves had positive fecal samples, it is possible that a proportion of these samples were not due to active shedding of MAP caused by an infection, but rather the result of passive shedding from exposure to the contaminated environment. It was reported that a higher prevalence of MAP caused more passive shedding events, due to increased environmental contamination [41]. Shedding ceased when CE calves were individually housed in a clean environment; and this may indicate that the shedding detected in group housing was due to passive shedding caused by ingestion of contaminated feces in the environment [26]. However, the decrease in frequency of sampling at that time may have also accounted for this lack of positive fecal samples, due to the frequency of intermittent shedding detected during group housing. It is noteworthy that decreases in calf fecal shedding at 4 months, and as early as 2 months, were reported in experimental trials [15, 22, 23, 37, 42], making it impossible to resolve the nature of this shedding.

Both IN and CE calves shed intermittently in our study, which was detected due to frequent fecal sampling. Others have reported intermittent shedding; however, the interval between positive samples largely depended on the interval between samplings [15, 43]. In the current study, positive fecal samples were followed by negative fecal samples for anywhere from 2 days to 5 weeks before another positive sample was detected. These findings may have large implications for sampling calves on farm and/or incorporating calf sampling into control programs, as calves may shed MAP 1 day, yet cease to be positive on following days/weeks. This creates narrow intervals for detection of potentially infectious young stock that may introduce new infections to pen-mates.

In addition to fecal testing for diagnosis, immune responses are also used to diagnose infected animals [44]. All calves were ELISA-negative for the duration of the experimental trial. This was not surprising, as the main limitation of the antibody ELISA is the ability to detect early stages of infection due to the humoral response being related to the severity of infection [9, 45, 46]. Additionally, the earliest that infection trials with similar doses detected positive antibody responses was 4 months after exposure [28, 33, 37, 47, 48]. The INF-γ immune response is generally a more sensitive indication of early infection or indication that an animal has been exposed to MAP [9, 46]; however, concerns regarding interpretation of the test [32, 49], as well as high individual variability [31] indicate the need for guarded interpretation and further optimization. Among IN calves, consecutive INF-γ positive samples began as early as 43 days after inoculation, and consecutive positive samples continued for all IN calves until euthanasia. Interestingly, of those CE calves with an INF-γ response, it was first detectable at 33 days of group housing (first exposure) or sooner (Calf 17), which indicates that they had a quicker cellular immune response than in IN calves (55 days). It has been reported that a lower dose of antigen may lead to a faster, more effective response [50]. Furthermore, a lower dose of MAP given over a longer interval (trickle dose) may lead to an earlier cellular immune response [28]; however, further research is needed.

All 4 control calves had a positive fecal sample 21 days after the start of group housing, and 1 calf had an additional positive fecal sample on day 56, as well as 2 positive INF-γ samples. It is not unusual to detect positive INF-γ samples among non-infected control calves [51]. All control calves were MAP tissue culture-negative. Despite the 5 positive fecal samples collected from calves in the control pen, all environmental samples collected for the duration of the study were negative. Although the CT threshold is high, resulting in a high specificity of fecal culture to identify true negative samples, control fecal samples collected on day 21 all had CT values well below the cut-off. Perhaps passive shedding of MAP on d 21 resulted from transmission (via an object or air) from an experimental pen in the barn, or samples were contaminated on the day of sampling. However, this was unlikely due to the stringent protocols and strict biosecurity measures in place. It is unlikely that any control calves became infected, based on negative results for tissues, fecal and environmental samples during the trial, and it is possible these samples became positive during processing in the laboratory, as all control animals tested positive on the same day.

In conclusion, this study provided strong evidence that CE calves can become infected with MAP, and are at risk for transmission from infectious calves in group pens. It was noteworthy that 50% of CE calves had MAP-positive tissue results after 3 months of group housing, 5 (36%) had evidence of a cellular immune response, and all had MAP-positive fecal samples (indicating shedding of bacteria). Transmission among group-housed calves is currently largely overlooked in current control programs, but based on evidence from the current study, calf-to-calf transmission may be a source of new infections within a herd. Although there are still important knowledge gaps in the field regarding pathogenesis, progression, and recovery among infected animals, potential transmission among group-housed calves should be considered in JD control and prevention programs.

## Abbreviations

CE: contact-exposed
BHI: brain heart infusion
HPC: hexadecylpyridinium chloride
IN: inoculated
LN: lymph node
JD: Johne’s disease
MAP: *Mycobacterium avium* subspecies *paratuberculosis*
